# Switching from premixed insulin to glargine-based insulin regimen improves glycaemic control in patients with type 1 or type 2 diabetes: a retrospective primary care-based analysis

**DOI:** 10.1186/1475-2840-8-9

**Published:** 2009-02-16

**Authors:** Peter Sharplin, Jason Gordon, John R Peters, Anthony P Tetlow, Andrea J Longman, Philip McEwan

**Affiliations:** 1 CHKS Health Economics Unit, Health Park, Cardiff, UK; 2 Department of Medicine, School of Medicine, Cardiff University, Cardiff, UK

## Abstract

**Background:**

Insulin glargine (glargine) and premixed insulins (premix) are alternative insulin treatments. This analysis evaluated glycaemic control in 528 patients with type 1 (n = 183) or type 2 (n = 345) diabetes, after switching from premix to a glargine-based regimen, using unselected general practice (GP) data.

**Methods:**

Data for this retrospective observational analysis were extracted from a UK GP database (The Health Improvement Network). Patients were required to have at least 12 months of available data, before and after, switching from premix to a glargine-based regimen. The principal analysis was the change in HbA_1c _after 12 months of treatment with glargine; secondary analyses included change in weight, bolus usage and total daily insulin dose. Inconsistent reporting of hypoglycemic episodes precludes reliable assessment of this outcome. Multivariate analyses were used to adjust for baseline characteristics and confounding variables.

**Results:**

Both cohorts showed significant reduction in mean HbA_1c _12 months after the switch: by -0.67% (p < 0.001) in the type 1 cohort and by -0.53% (p < 0.001) in the type 2 cohort (adjusted data). The size of HbA_1c _improvement was positively correlated with baseline HbA_1c_; patients with a baseline HbA_1c _≥ 10% had the greatest mean reduction in HbA_1c_, by -1.7% (p < 0.001) and -1.2% (p < 0.001), respectively. The proportion of patients receiving co-bolus prescriptions increased in the type 1 (mean 24.6% to 95.1%, p < 0.001) and type 2 (mean 16.2% to 73.9%, p < 0.001) cohorts. There was no significant change in weight in either cohort. Total mean insulin use increased in type 2 diabetes patients (from 0.67 ± 1.35 U/Kg to 0.88 ± 1.33 U/Kg, p < 0.001) with a slight decrease in type 1 diabetes patients (from 1.04 ± 2.51 U/Kg to 0.98 ± 2.58 U/Kg, p < 0.001).

**Conclusion:**

In everyday practice, patients with type 1 or type 2 diabetes inadequately controlled by premix insulins experienced significant improvement in glycaemic control over 12 months after switching to a glargine-based insulin regimen. These findings support the use of a basal-bolus glargine-based regimen in patients poorly controlled on premix.

## Background

Achieving the recommended target for glycaemic control (glycated haemoglobin [HbA_1c_] 6.5% to 7.0%) [1-7] in patients with type 1 or type 2 diabetes is essential for reducing the risk of serious diabetes-related complications [8-10]. In type 1 diabetes this can only be achieved with insulin therapy. For patients with type 2 diabetes, insulin therapy is indicated after failure to achieve glycaemic control despite increasingly aggressive treatment with oral antidiabetic drugs (OADs) that are prescribed in combination with lifestyle changes.

Of the available insulin preparations, premixed insulins (premix), combine fixed ratios of short- and intermediate acting insulins into a single formulation that are generally injected once or twice daily. In general, premix insulins do not mimic physiologic insulin profiles and a substantial proportion of patients have sub-optimal glycaemic control [11]. Insulin glargine (glargine, Lantus^®^), a long-acting basal insulin analogue, available in the UK since 2002, has a prolonged and predictable absorption rate over 24 hours, without peak effects [12]. In insulin-naïve patients with type 2 diabetes, glargine treatment combined with OADs is associated with significantly lower HbA_1c _levels and fewer episodes of symptomatic hypoglycaemia compared with premix [13].

For patients who are inadequately controlled with premix, switching to a glargine-based regimen may offer advantages in terms of glycaemic control, tolerability and patient satisfaction with treatment [14-16]. In a retrospective sub-analysis of the AT.LANTUS (A Trial comparing Lantus Algorithms to achieve Normal blood glucose Targets in subjects with Uncontrolled blood Sugar with type 2 diabetes mellitus) study [16], including 686 patients with type 2 diabetes taking premix at baseline, poorly controlled patients who switched to glargine ± OADs/prandial insulin showed significantly improved glycaemic control and a low incidence of severe hypoglycaemia after 6 months on treatment. Consistent with these findings, a 12-week observational study [14] showed that patients with type 2 diabetes who switched from premix to glargine plus OAD showed significant improvement in mean HbA_1c _(-1.1 ± 1.0%, p ≤ 0.001) and reduction in body weight (-1.5 ± 3.3 kg, p ≤ 0.001). However, whether these benefits extend to both type 1 and type 2 diabetes patients in routine clinical practice has yet to be investigated. Therefore, this retrospective analysis was performed to evaluate the effect of switching from premix to a glargine-based regimen on glycaemic control, body weight and insulin use in patients with type 1 or type 2 diabetes in a daily practice setting.

## Methods

### Data source

The data were sourced from a large national computerised medical record database known as The Health Improvement Network (THIN), which includes data from 211 UK primary care practices collected over a 15 year period from about 5 million patients, of whom 2.34 million were actively registered with a practice and prospectively followed [17]. The THIN database is not supported by any industrial sponsor, nor biased towards any particular disease group. THIN data on patient demographics, medical history, test results and drug treatments are collected in a non-interventional manner during daily record keeping within the general practice. To ensure confidentiality of patient information, the data are anonymised at the collection stage using encrypted identifiers for the physician and individual.

From data collected between July 2002 and December 2005, as described previously [18,19], 137,258 patients were identified as diabetic based on a relevant medical diagnosis using the Read code system [20] or prescription of OADs. Diagnosis of diabetes was attributed in a stepwise manner. For those few patients who did not have a specific diagnosis of diabetes (but use of insulin), a diagnosis of type 2 diabetes was attributed if the patient had received any non-insulin, diabetes-related medication, otherwise type 1 diabetes was assumed. Overall, 90% of patients were identified as having a diagnosis of type 2 diabetes. Ethical approval for this analysis was obtained from the London Multiple Research Ethics Committee (Number 06/MRE02/32) before commencing data extraction.

Individuals were included in the current analysis if a) they had been prescribed premix for at least 12 months before switching to glargine and b) they continued glargine without a switch to another basal insulin for at least 12 months. Following a switch to glargine patients could receive OADs and/or boluses of prandial insulin in addition to basal glargine. Information on associated comorbidities including myocardial infarction, stroke, peripheral vascular disease, neuropathy, nephropathy, and retinopathy was also extracted. Use of analogue and human prandial insulins could not be distinguished from information collected.

### Design and outcome measures

This was a retrospective, 24-month, non-randomised analysis. The principal analysis was glycaemic control measured using HbA_1c_. Measurements were performed locally in each centre and mean HbA_1c _values were calculated every 3 months before and after switching insulin therapy using actual or linearly interpolated values. Although much of the UK is currently HbA_1c _DCCT-aligned, and primary care practices use National Health Service hospital laboratories which are members of quality assurance schemes, the degree of standardisation at the time of data collection (2002–2006) is not known. However, our study depends on change in HbA_1c _and will thus be less sensitive to differences in calibration between assays. Secondary analyses included mean change in weight (kg) calculated as for HbA_1c_, mean change in prescribed daily insulin dose calculated as units prescribed divided by the number of days covered by the prescription, the proportion of patients using bolus prandial insulin, and the percentage of patients achieving defined HbA_1c _levels. Self-reported episodes of hypoglycaemia were recorded by general practitioners during each 3 monthly interval.

### Statistical methods

Linear interpolation of missing data was performed where a patient had at least 2 data measurements during each 12 month period (prior to and following switch) and data was not missing during two consecutive 3 monthly intervals. Unadjusted results for the principle (HbA_1c_) and secondary analyses used linearly interpolated data and were summarised using descriptive statistics. For the unadjusted results the mean change during the 12 month prior to and following the switch was calculated. Comparisons were performed using paired t-tests. Graphical analyses were based on linearly interpolated data, which provides a clearer graphical interpretation of the results.

For the principle analysis of change in HbA_1c _a multivariate analysis using actual patient data was performed. Actual patient data was preferred over interpolated values; multivariate models constructed using the later showing no appreciable effect on the model specification or statistical inference. Data was evaluated using multiple linear mixed regression analyses, adjusting for repeated measures per patient over time, with change in HbA_1c _relative to time of insulin initiation as the dependent variable with the following pre-defined (fixed-effects) exploratory covariates; age, weight, sex, type of diabetes, number of OADs used before commencing insulin, number of OADs used in combination with insulin at initiation, disease duration, presence of hypoglycaemia and associated co-morbidities during the study.

Multivariate models were developed with SPSS for Windows (version 8; SPSS, Chicago, IL, USA) using a backward stepwise approach; non-significant variables at the 5% level were excluded. Sensitivity analyses were performed to investigate the effect of baseline HbA_1c _levels on treatment efficacy. Secondary endpoints and the percentage of patients achieving set HbA_1c _levels were summarised descriptively.

## Results

### Subjects and baseline characteristics

A total of 528 patients, 183 (35%) with type 1 diabetes and 345 (65%) with type 2 diabetes, were included in the analysis (Table 1). Mean HbA_1c _at baseline before switching was similar in each group (9.4% and 9.3%, respectively). Overall, 39% of patients with type 1 diabetes and 21% with type 2 diabetes had received bolus insulin doses in the previous 12 months prior to the switch, whilst 0% and 38% of patients with type 1 and type 2 diabetes respectively were taking an OAD.

**Table 1:** Baseline characteristics of patients switching from premix to glargine

	**Type 1 diabetes**	**Type 2 diabetes**
n (%)	183 (35)	345 (65)
% male	53.0	53.0
Age (years)*	22.9 ± 15.5	55.8 ± 16.6
Weight (kg)*	67.7 ± 2.7	85.3 ± 2.2
Baseline HbA_1c _*†	9.4 ± 1.6	9.3 ± 1.5
Duration of diabetes (years)‡	7.9 ± 8.7	8.1 ± 6.7
Number of co-morbidities§	2.5 ± 2.4	2.4 ± 1.9
Premix insulin only (%)	61%	48%
Premix insulin with OAD (%)	0%	38%
Premix + short acting insulin (%)	25%	16%
Premix + basal insulin (%)	8%	5%
Premix + short acting + basal insulin (%)	2%	3%
Premix + other insulin +/-OADs (%)	0%	2%
OADs per patient before starting insulin therapy*¶	-	0.4 ± 0.5
Hypoglycaemia episodes in 3 month period*||		
No. episodes	38	72
Mean no. episodes per patient	0.21	0.21

OAD = oral antidiabetic drug; HbA_1c _= glycated haemoglobin. Data are mean ± SD unless indicated.

*Significant variables investigated in multivariate analysis.

†Data missing for 29 patients with type 1 diabetes and 27 patients with type 2 diabetes.

‡Data missing for 2 patients with type 1 diabetes and 5 patients with type 2 diabetes.

§Includes myocardial infarction, stroke, peripheral vascular disease, neuropathy, nephropathy and retinopathy.

¶Number of oral diabetic treatments (e.g. metformin, sulfonylureas) prescribed prior to commencing insulin.

||Hypoglycaemic episodes reported during the 3 month period prior to switch. Episodes reported in 27 patients with type 1 diabetes and 44 patients with type 2 diabetes.

### Change in HbA_1c_

**Figure 1:**
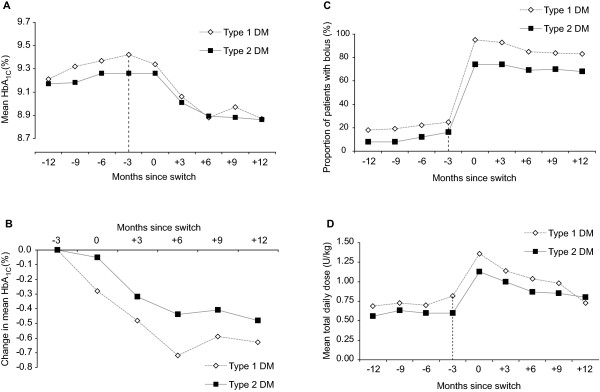
**Mean HbA_1c _12 months before and after switching from premix to glargine (A), mean change in HbA_1c _after switch (B), mean use of bolus insulin before and after switch (C) and mean total daily insulin dose before and after switch (D) (unadjusted data)**. The last measurement for premix is at -3 months (indicated by vertical dotted line). During period -12 m to -3 m patients are taking premix only. During the 3 month switch time point (0 months) patients may be prescribed premix and glargine. During period +3 m to +12 m patients are only prescribed glargine (± prandial boluses). Linearly interpolated data were used to graphically depict the change in each parameter. Linearly interpolated data affords a clearer graphical interpretation but may bias estimates of variance; as such error bars (95% confidence intervals for the means) are not reported. Total daily insulin dose was calculated according to the number of units prescribed divided by the number of days covered by the prescription.

Mean HbA_1c _increased in the 12 months prior to the switch in both diabetic cohorts, by 0.21% (from 9.21% to 9.42%, p > 0.05) in the type 1 cohort and by 0.05% (9.17% to 9.26%, p > 0.05) in the type 2 cohort (Figure 1A). 12 months after the switch, mean HbA_1c _was significantly lower compared with baseline in each cohort, decreasing by 0.63% (from 9.42% to 8.79%, p = 0.003) in patients with type 1 diabetes and by 0.47% (from 9.26% to 8.79%, p = 0.0004) in patients with type 2 diabetes (Figure 1B). The greatest decrease in mean HbA_1c _was observed in the first 6 months following the switch to a glargine-based regimen. After adjustment for significant demographic and clinical covariates, including age, weight, baseline HbA_1c_, hypoglycaemia and concomitant use of OADs, the reduction in mean HbA_1c _over 12 months of glargine treatment was 0.67% (p < 0.001) in the type 1 diabetes cohort and 0.53% in the type 2 diabetes cohort (p < 0.001) (Table 2). Sensitivity analyses showed that improvement in HbA_1c _after switching to glargine was positively correlated with baseline HbA_1c_. Patients with baseline HbA_1c _≥ 10% had the greatest reduction in mean HbA_1c _(-1.7% in the type 1 diabetes cohort and -1.2% in the type 2 diabetes cohort) (Table 2). In each cohort, the reduction in HbA_1c _did not differ significantly by sex, age or weight (data not shown).

**Table 2:** Adjusted HbA_1c _reduction over 12 month period in patients switching from premix to glargine*

**Variable**	**Type 1 (n = 183)**			**Type 2 (n = 345)**		
		
	**No**.	**Δ HbA_1c _(%)**	**p-value**†	**No**.	**Δ HbA_1c _(%)**	**p-value**†
Overall	183	-0.67	<0.001	345	-0.53	<0.001
By baseline HbA_1c _level						
≥ 7%	151	-0.67	<0.001	306	-0.56	<0.001
≥ 8%	128	-0.80	<0.001	255	-0.68	<0.001
≥ 9%	86	-1.06	<0.001	169	-0.84	<0.001
≥ 10%	46	-1.74	<0.001	88	-1.20	<0.001

OAD = oral antidiabetic drugs; HbA_1c _= glycated haemoglobin.

*Adjusted for demographic and clinical covariates including age, weight, hypoglycaemia, concomitant use of OADs and baseline HbA_1c _(in the 3 months prior to insulin initiation).

†p-values by the paired t-test for difference in mean HbA_1c _following switch from premix to glargine.

### Proportion of patients reaching HbA_1c _levels

Overall, 35% of patients achieved a HbA_1c _level of 7% within 12 months of the switch. 32% of the type 1 cohort and 33% of the type 2 cohort achieved a reduction in HbA_1c _≥ 1%.

### Episodes of hypoglycaemia

During the 12 months prior to the switch from premix to glargine, 105 hypoglycaemic episodes were reported by the patients with type 1 diabetes (0.57 episodes per patient/year), while 207 episodes were reported by patients with type 2 diabetes (0.60 episodes per patient/year). After switching to glargine, the respective data were 194 episodes for the patients with type 1 diabetes (1.06 episodes per patient/year) and 406 episodes for the patients with type 2 diabetes (1.18 episodes per patient/year) during the following 12 months.

### Change in use of boluses, total insulin usage and weight

The majority of patients switched from premix to a basal-bolus glargine regimen. Significant increases in the mean use of bolus regimens in both diabetic cohorts were recorded (from 24.6% on premix to 95.1% on glargine in the type 1 cohort [p < 0.001], and from 16.2% to 73.9% in the type 2 cohort [p < 0.001], Figure 1C). Switching from premix to glargine was associated with an increase in the mean total (glargine + prandial) daily insulin dose in the type 2 diabetes cohort (from 0.67 ± 1.35 U/Kg to 0.88 ± 1.33 U/Kg, p < 0.001) but a slight decrease in the type 1 cohort (from 1.04 ± 2.51 U/Kg to 0.98 ± 2.58 U/Kg, p < 0.001) (Figure 1D). The small initial increase in the first 6 months after the switch in each cohort may be due a "stock building effect", whereby patients are initially prescribed additional insulin doses to allow them to store a security stock when starting the treatment with their new insulin. The use of OADs remained unchanged after the switch, with quarterly estimates indicating that between 34% and 37% of patients received OADs. There was no significant change in weight in the type 2 cohort (mean +0.3 kg, from 85.3 ± 2.2 kg to 85.6 ± 2.6 kg, p > 0.05) with a moderate increase in type 1 patients (mean +3.7 kg, from 67.6 ± 2.7 kg to 71.3 ± 3.5 kg, p > 0.05).

## Discussion

The results from this analysis show that in everyday clinical practice, switching to a basal-bolus glargine-based insulin regimen improves glycaemic control in patients with type 1 or type 2 diabetes inadequately controlled on a premix-based insulin regimen. The overall mean decrease in HbA_1c _was 0.67% among type 1 patients and 0.53% among type 2 patients observed 12 months after switching to a glargine-based regimen.

These findings are supported by data from other observational studies in patients with type 2 diabetes switching from premix to glargine-based regimen [14-16] and by a randomised comparison of a premix-based regimen versus a glargine-based regimen in type 2 diabetic patients previously treated with a glargine-based regimen plus OADs [21]. In this later study, the difference in HbA_1c _change after 6 months of treatment was 0.22% in favour of the glargine-based regimen, approximately half the decrease noted after 12 months in the type 2 patient cohort for our study.

Additionally, as far as we are aware, this analysis also provides the first evidence of a similar benefit in patients with type 1 diabetes. Sensitivity analyses showed that improvement in HbA_1c _was greatest in patients with the poorest glycaemic control at baseline (mean increase of 1.2% to 1.7% in patients with baseline HbA_1c _levels ≥ 10%), with the magnitude of improvement equivalent to that observed in studies of patients newly commencing insulin therapy [22]. The United Kingdom Prospective Diabetes Study showed that a 1% reduction in HbA_1C _was associated with a 14% reduction in myocardial infarction, a 14% reduction in all-cause mortality and a 37% reduction in microvascular complications [23]. Therefore, the reduction in HbA_1C _of 0.5 to 0.7% observed in the overall population and ≥ 1% in the sensitivity analysis, achieved by switching to an insulin glargine-based regimen can be considered clinically meaningful as these results may translate to clinical outcomes benefits in the longer-term.

A number of features of this analysis strengthen our findings. Patients included in the analysis had frequent (at least 3-monthly) follow-up assessment over an extended period (12 months) before and after switching. Glycaemic control was evaluated using a uniform, valid, reliable and widely used measure (HbA_1c_), which is of relevance to a real-life clinical practice. Additionally, analysis of changes in clinical outcomes was assessed by means of linear interpolation for missing data and multiple regression techniques to account for factors that may influence the change in HbA_1c_. In particular, the multivariate model attempted to account for the significant increases in the use of bolus regimens in patients receiving glargine.

However, we do acknowledge a number of limitations. First, we recognise that retrospective observational studies do not provide the same robust level of evidence as randomised controlled trials. Countering this, it should be noted that results from such randomised evaluations may not translate readily to daily practice for a number of reasons [24,25]. Patients treated within clinical trials generally receive a higher standard of care than that provided in daily clinical practice [26,27] and they typically have restricted inclusion criteria in selected populations which are generally not representative of those patients treated in clinical practice. Moreover, findings from a single randomised controlled trial can be subject to substantial population bias which can skew results. These points argue for the use of observational studies (such as the current analysis), which are generally well equipped to describe actual health outcomes in a real-life clinical setting. Health technology assessment bodies, including the National Institute of Clinical Excellence in the UK, increasingly seek data outside the setting of randomised controlled trials [28]. However, we do acknowledge that the level of data collection in our analysis did not permit investigation of various factors that may have influenced our findings, including certain background characteristics (e.g. ethnicity, body mass index) of the patient population [29-31], dosing regimen (once vs. twice daily) [32] increased patient compliance following the switch (e.g. higher number of general practice visits) and timing of administration of glargine (morning vs. bedtime) [33,34]. The low number of patients included in the study did not permit certain subgroup analyses such as the influence of prandial boluses on glycaemic control in type 2 patients switched onto a glargine-based insulin regimen [16].

Second, it was not possible to reliably assess data concerning hypoglycaemic episodes in patients who switch from a premix-based regimen to a glargine-based regimen. A lack of consistency in recording these data in the THIN database meant that there is strong likelihood that we underestimated the real incidence of hypoglycaemia and only captured the most severe episodes. The incidence of hypoglycaemia may also reflect the self-reporting methods used, as patients and physicians were not requested to provide specific details of each episode. The limitations of the database also did not allow assessment of the severity or nature (e.g. nocturnal) of hypoglycaemic episodes before and after the switch. A higher number of hypoglycaemic episodes were noted after the switch from premix to glargine. Should this trend be real, the significant improvement in glycaemic control observed with glargine in both diabetic cohorts and the higher use of bolus regimens in both diabetic cohorts is likely to make patients more susceptible to episodes of hypoglycaemia. The reason for the slightly higher mean number of hypoglycaemic episodes per patient observed in the type 2 cohort compared with type 1 diabetic cohort is not known and not in keeping with previous observations.

Third, the decision to switch insulin treatment was not based on a standard treatment algorithm but instead on the clinical judgement of individual clinicians, thereby introducing a subjective bias in the management of patients in each cohort. Furthermore, as data in this analysis were included from a large number of general practice units, this may had led to further heterogeneity in the data. However, it is possible that the extended period of assessment (12 months) before and after the switch may have limited the potential for introduction of bias in our analysis. In respect of this point, it is reassuring that our findings are supported by other analyses from different populations and geographic locations using different methods of collection of information [14-16]. Finally, as with any observational study there may be concerns about missing data. In the current study, interpolated HbA_1c _data were available for 87% of patients during the months preceding and immediately following the switch, decreasing to 65% of patients during the 12 months after the switch. Overall, interpolated HbA_1c _data was used in 60% of patients. However, the main analysis performed in our study was based on the adjusted change in HbA_1c _which only used actual and complete data values.

While findings from this analysis suggest that the use of an glargine-based regimen possibly combining glargine with either OADs or prandial insulin may be a useful alternative for improving glycaemic control when other insulin therapies have failed, there is evidence that glycaemic control is still suboptimal in UK general practice. In our analysis, two-thirds of patients remained above a HbA_1c _level of 7%, indicating that a large proportion of patients do not reach preferred target levels. This could indicate that the initiation of insulin was possibly too late and that additional treatment strategies are needed. This may include more aggressive titration with insulin glargine (in the current analysis glargine treatment had no significant effect on weight despite a moderate increase for the type 1 patients suggesting suboptimal treatment), greater use of OAD therapy in patients with type 2 diabetes (only 38% were receiving OAD therapy at baseline and 35% at 12 months), additional doses of prandial insulin and the use of educational programs [16,35-37].

## Conclusion

This retrospective analysis shows that switching from premix to a basal-bolus glargine-based insulin regimen improves glycaemic control substantially. Given the caveats associated with retrospective data collection, our findings suggest that this approach may be useful in a clinical practice setting for the management of patients in whom premix is suboptimally effective or poorly tolerated.

## Abbreviations

HbA_1c_: glycated haemoglobin; OADs: oral antidiabetic drugs; THIN: The Health Improvement Network.

## Competing interests

All authors were consultants to Sanofi-Aventis, had full access to all the data in the study and took responsibility for the decision to submit for publication.

## Authors' contributions

PS and JG performed the statistical analyses and drafted the manuscript. JRP, APT, AL, and PM were involved in study design, coordination and data acquisition. All authors have read and approved the final manuscript.
